# Lanthanum(III) triggers AtrbohD- and jasmonic acid-dependent systemic endocytosis in plants

**DOI:** 10.1038/s41467-021-24379-z

**Published:** 2021-07-15

**Authors:** Mengzhu Cheng, Lihong Wang, Qing Zhou, Daiyin Chao, Shingo Nagawa, Ding He, Jiazhi Zhang, Hui Li, Li Tan, Zhenhong Gu, Xiaohua Huang, Zhenbiao Yang

**Affiliations:** 1 National and Local Joint Engineering Research Center of Biomedical Functional Materials, Jiangsu Collaborative Innovation Center of Biomedical Functional Materials, School of Chemistry and Materials Science, Nanjing Normal University, Nanjing, China; 2 State Key Laboratory of Food Science and Technology, Jiangnan University, Wuxi, China; 3 National Key Laboratory of Plant Molecular Genetics, Institute of Plant Physiology and Ecology, Shanghai Institutes for Biological Sciences, Chinese Academy of Sciences, Shanghai, China; 4 Fujian Agriculture and Forestry University-University of California, Riverside, Joint Center for Horticultural Biology and Metabolomics, Haixia Institute of Science and Technology, Fujian Agriculture and Forestry University, Fuzhou, China; 5 Shanghai Center for Plant Stress Biology, Shanghai Institute of Biological Sciences, Chinese Academy of Sciences, Shanghai, China; 6 School of Life Sciences, East China Normal University, Shanghai, China; 7 Department of Botany and Plant Sciences, Center for Plant Cell Biology, Institute of Integrative Genome Biology, University of California, Riverside, CA USA

**Keywords:** Cell signalling, Plant cell biology

## Abstract

Trivalent rare earth elements (REEs) are widely used in agriculture. Aerially applied REEs enter leaf epidermal cells by endocytosis and act systemically to improve the growth of the whole plant. The mechanistic basis of their systemic activity is unclear. Here, we show that treatment of *Arabidopsis* leaves with trivalent lanthanum [La(III)], a representative of REEs, triggers systemic endocytosis from leaves to roots. La(III)-induced systemic endocytosis requires AtrbohD-mediated reactive oxygen species production and jasmonic acid. Systemic endocytosis impacts the accumulation of mineral elements and the development of roots consistent with the growth promoting effects induced by aerially applied REEs. These findings provide insights into the mechanistic basis of REE activity in plants.

## Abstract (web summary)

Rare earth elements are widely used in agriculture to promote plant growth. Here the authors show that aerial application of a rare earth element to the leaves triggers a graft-transmissible, RbohD- and jasmonic acid-dependent systemic signal that triggers endocytosis and mineral nutrient uptake in roots.

## Introduction

Trivalent rare earth elements (REEs) have long been known to promote plant growth and are widely used as growth regulators to improve crop productivity mainly via foliar application^1–3^. Interestingly, foliar application of REEs induces systemic impacts on plant growth and development (such as changes in leaf area and weight, root length and weight) and physiological activities (such as changes in photosynthesis in leaves and mineral element levels in roots), which are commonly observed 7 days and 24 h after the application of REEs, respectively^2,4,5^. In addition, REEs are widely used materials in industry, medicine, military, etc.^6–8^. Due to the ever-increasing usage, REEs increasingly accumulate in the environment and living organisms^9^, raising significant concerns for human health^9–12^. Thus, REEs have become important elements that affect living organisms^9–12^. However, the modes of REE activity in living organisms have been puzzling researchers over the past 100 years. In particular nothing is known about the cellular and molecular mechanisms by which locally applied REEs trigger a systemic response in plants^4,13–15^. Recently, our studies using interdisciplinary approaches, such as electron microscopy autoradiography of REE radioisotopes, including lanthanum [^140^La(III)], cerium [^141^Ce(III)], and terbium [^160^Tb(III)], and total internal reflection fluorescence microscopy, have demonstrated that aerially applied REEs for 12 h initiate their own endocytosis in plant leaf cells^4,14,15^. Such endocytosis has not been observed in plants treated with other metal elements^4^. The unique effects of REEs have drawn a great deal of attention from scientists in various fields^16–23^. However, the mechanisms for the systemic responses in plants triggered by aerial REE application remain unclear.

In this study, we find that La(III) application on *Arabidopsis* leaves activates the production of systemic signal(s) inducing endocytosis in roots and that this systemic response requires coordinate action of AtrbohD (an NADPH oxidase) with jasmonic acid (JA). Systemic endocytosis changes the accumulation of mineral elements in roots and the growth of the whole plant, including leaf expansion, primary root growth, and lateral root formation. These findings have demonstrated the existence of systemic endocytosis, suggested a mechanistic basis for systemic signaling mediated by the coordinate action of AtrbohD and JA, and created opportunities for the study of systemic signaling and the mode of REE activity in living organisms.

## Results

### La(III) induces systemic endocytosis

We used several complementary methods to visualize endocytosis induced by aerially applied 30 or 80 μM LaCl_3_ that has a respective promotive or inhibitory effect on the growth and physiological activities of the whole *Arabidopsis* plant (Supplementary Table 1). First, we confirmed LaCl_3_-induced endocytosis in leaf epidermal cells stained with *N*-(3-triethylammoniumpropyl)-4-(4-diethylaminophenylhexatrienyl) (FM4-64), a fluorescent dye that only penetrates cell membranes via endocytosis^24,25^, as previously reported^4,15^. Treatment of wild-type *Arabidopsis* (Col-0) leaves with 30 µM LaCl_3_ greatly increased the frequency of endocytosis (the percentage of cells exhibiting endocytosis) and the number of endocytic vesicles per cell (the average number of endocytic vesicles, i.e., dot- or circle-like structures stained with FM4-64) in leaf epidermal cells (Supplementary Fig. 1a, b), respectively. Next, transmission electronic microscopy (TEM) was used to observe the sizes of 30 µM LaCl_3_-induced vesicles and their likely biogenesis because TEM can determine the morphology of endocytic vesicles with high resolution^26^. The TEM images showed that these vesicles varied in sizes mainly around 100–200 nm in diameters localized to the vicinity of the plasma membrane (PM) and appeared to result from the PM invagination (Supplementary Fig. 1c, d). Treatment with 80 µM LaCl_3_ induced more pronounced changes in vesicle formation (Supplementary Fig. 1). In mock-treated epidermal cells, we did not observe such vesicles and the PM invagination (Supplementary Fig. 1). These results are consistent with our previous results observed via electron microscopy autoradiography of ^140^La(III) radioisotopes^4,15^.

Because the majority of endocytic events reported in plant cells are clathrin-mediated endocytosis (CME)^24,27^, we tested whether LaCl_3_-induced endocytosis in leaf epidermal cells was also clathrin-mediated using a line expressing green fluorescent protein (GFP)-tagged clathrin light chain 1 (CLC1, an essential subunit of clathrin coats^28^) and by co-staining with FM4-64. We found that CLC1-GFP-labeled vesicles were only observed in the epidermal cells of leaves treated with 30 or 80 µM LaCl_3_ (Supplementary Fig. 2a). CLC1-GFP showed overlapping distribution with FM4-64 both on the PM and the membrane of LaCl_3_-induced vesicles (Supplementary Fig. 2a). These results suggest that LaCl_3_ treatment induced CME in leaf epidermal cells. The frequency of CME and the number of CLC1-GFP-labeled vesicles per cell in leaves treated with 80 µM LaCl_3_ were much higher than in those treated with 30 µM LaCl_3_ (Supplementary Fig. 2b). Importantly, time-lapse imaging showed that LaCl_3_ treatment induced the budding of CLC1-GFP-labeled membrane and their pinching off from the PM in leaf epidermal cells (Supplementary Movie 1). Taken together, our results demonstrate that LaCl_3_ treatment on *Arabidopsis* leaves induces CME in leaf epidermal cells. This conclusion is further supported by inhibiting CME using genetic approaches (see below). It is noteworthy that some FM4-64- or CLC1-GFP-labeled vesicles >200 nm were observed in confocal laser scanning microscope (CLSM) images (Supplementary Figs. 1a and 2a), which may result from the fusion of vesicles (Supplementary Movie 1) and/or lower spatial resolution of CLSM^26^.

**Fig. 1:**
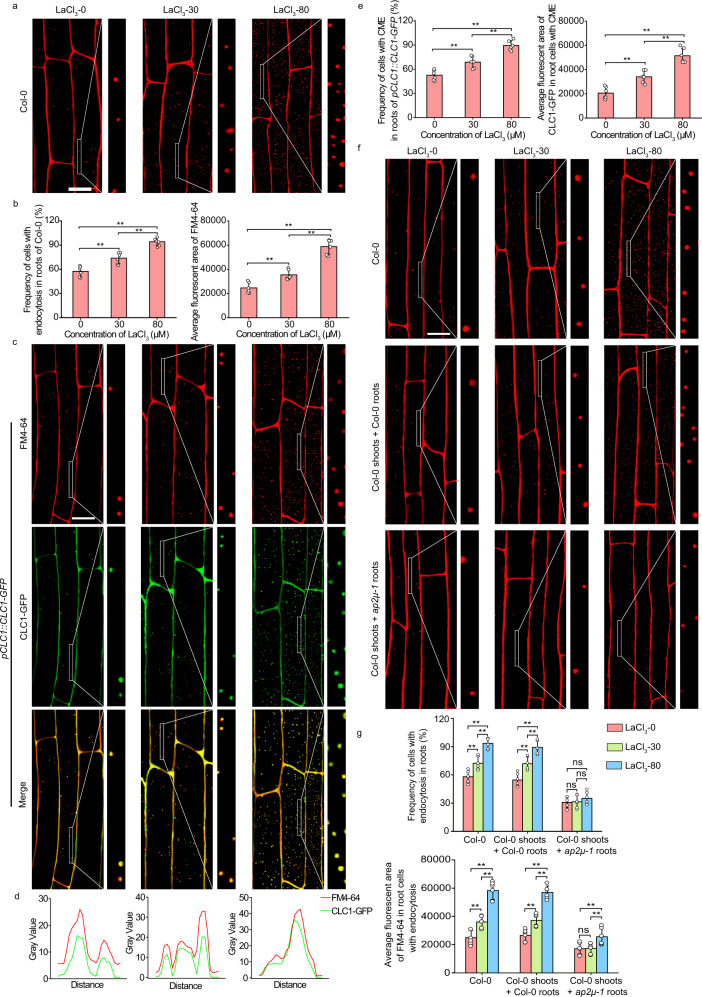
LaCl_3_ induced CME in root cells. **a** The representative CLSM images of Col-0 root cells. Leaves of Col-0 were treated with 0, 30, or 80 µM LaCl_3_ for 12 h and then roots were stained with FM4-64. Bar = 10 μm. **b** Quantitative analysis of the frequency of root cells with endocytosis and average fluorescent area of FM4-64 in Col-0 root cells (data come from **a**). **c** The representative CLSM images of *pCLC1::CLC1-GFP* root cells. Leaves of *pCLC1::CLC1-GFP* were treated with 0, 30, or 80 µM LaCl_3_ for 12 h and then roots were stained with FM4-64. Bar = 10 μm. **d** The intensity plot of corresponding solid boxes shown in (**c**). **e** Quantitative analysis of the frequency of *pCLC1::CLC1-GFP* root cells with CME and average fluorescent area of CLC1-GFP in these cells (data come from **c**). **f** The representative CLSM images of Col-0, and the grafted Col-0 and *ap2μ-1* (shoots from Col-0 were grafted onto Col-0 and *ap2μ-1* roots) root cells. Leaves of all of these *Arabidopsis* were treated with 0, 30, or 80 µM LaCl_3_ for 12 h and then roots were stained with FM4-64. Bar = 10 μm. **g** Quantitative analysis of the frequency of root cells with endocytosis and average fluorescent area of FM4-64 in these cells of Col-0, and the grafted Col-0 and *ap2μ-1* (shoots from Col-0 were grafted onto Col-0 and *ap2μ-1* roots) root cells (data come from **f**). In **a**, **c**, and **f**, representative images from six independent measurements and three biological replicates (each replicate represents an independently treated plant) per measurement are represented, and the right sides of the CLSM images are the enlarged areas of solid boxes. In (**b**, **e**, **g**), values shown are means ± SEM, one-way ANOVA analysis with LSD multiple comparisons test (*n* = 6, **p < 0.01, n.s.: no significance).

It was reported that LaCl_3_-triggered endocytosis in plant leaves enhanced the contents of nutrient elements in leaf cells^4^. The nutrient elements of plants mainly come from their absorption from the soil by roots. Furthermore, endocytosis may contribute to the uptake of nutrient elements^29,30^. Thus, we speculated that the increased contents of nutrient elements in leaf cells could result from the increased endocytosis in root cells. We observed endocytosis in root epidermal cells 12 h after treatment of Col-0 leaves with LaCl_3_. Indeed, this treatment greatly enhanced endocytosis in root epidermal cells (Fig. 1a, b). Compared with the mock-treated control plants, the number of FM4-64-stained vesicles per root epidermal cell in 30 µM LaCl_3_-treated plants increased by 45% (Fig. 1a, b). Meanwhile, the frequency of root epidermal cells containing FM4-64-stained vesicles was 77% in 30 µM LaCl_3_-treated plants, compared to 54% of root epidermal cells in mock-treated control plants (Fig. 1a, b). Treatment of leaves with 80 µM LaCl_3_ induced a much greater increase in the frequency of cells with FM4-64-stained vesicles and the number of these vesicles per cell in the root epidermis (Fig. 1a, b). Moreover, CLC1-GFP exhibited overlapping distribution with FM4-64 both on the PM and vesicles in root epidermal cells (Fig. 1c–e). To assess whether the LaCl_3_-induced increase in FM4-64- and CLC1-GFP-co-labeled vesicles resulted from the enhanced CME in leaf epidermal cells or the inhibition of endosomal recycling in root epidermal cells, we next constructed the grafted plants in which shoots from Col-0 were grafted onto roots from the *ap2μ-1* mutant [a loss-of-function mutation in adaptor protein 2 (AP2), the CME-specific clathrin adapter^15,29^]. In these grafted plants whose leaves were treated with LaCl_3_, the number of FM4-64-stained vesicles in root epidermal cells was lower (Fig. 1f, g), compared with LaCl_3_-treated Col-0 plants or grafted control plants, in which Col-0 shoots were grafted onto Col-0 roots (Fig. 1f, g). These results suggest that LaCl_3_ treatment of leaves not only induces local CME in leaf epidermal cells but also systemically promotes CME in root epidermal cells.

Our previous study showed that LaCl_3_-induced endocytosis in leaf epidermis cells is not due to Cl^−^ or salt stress^4^. We next asked whether LaCl_3_-induced systemic endocytosis resulted from Cl^−^ or osmotic stress. To this end, we observed endocytosis in NaCl-treated Col-0 plants, in which the molar concentration of Cl^−^ in NaCl equaled to that in LaCl_3_. Twelve hours after treatment of leaves with NaCl, endocytosis in leaf and root epidermal cells was not changed, compared to the control cells (Supplementary Fig. 3). It was reported that salt stress often affects the growth and development of plant cells by changing osmotic pressure^31^, and osmotic stress can modulate the balance between exocytosis and CME^32^. To assess whether LaCl_3_ treatment caused osmotic stress in leaf epidermal cells, we measured osmotic pressure in Col-0 leaves and roots 12 h after LaCl_3_ treatment. Treatment of leaves with 30 or 80 μM LaCl_3_ for 12 h did not cause changes in osmotic pressure in either leaves or roots (Supplementary Fig. 4). Therefore, LaCl_3_-induced systemic endocytosis specifically results from La(III), but is not associated with Cl^−^ or osmotic stress.

### Systemic endocytosis induced by La(III) is independent of La(III) in roots

To assess whether the induction of systemic endocytosis could result from possible transport of La(III) from leaves to roots, we determined the levels of La(III) in leaves and roots 12 h after treatment of leaves with La(III) using inductively coupled plasma mass spectrometry (ICP-MS). We found the accumulation of La(III) in leaves (Supplementary Fig. 5), but did not detect La(III) in roots (Supplementary Fig. 5). This result suggests that 12 h after treatment of leaves with La(III), La(III) was not transported to roots, which is in agreement with our previous report that very little REEs move from leaves to roots 48 h after treatment of leaves with REEs^33^. Therefore, systemic endocytosis induced by La(III) is independent of La(III) in roots.

### La(III)-induced endocytosis in leaf cells triggers a systemic endocytosis signal

**Fig. 2:**
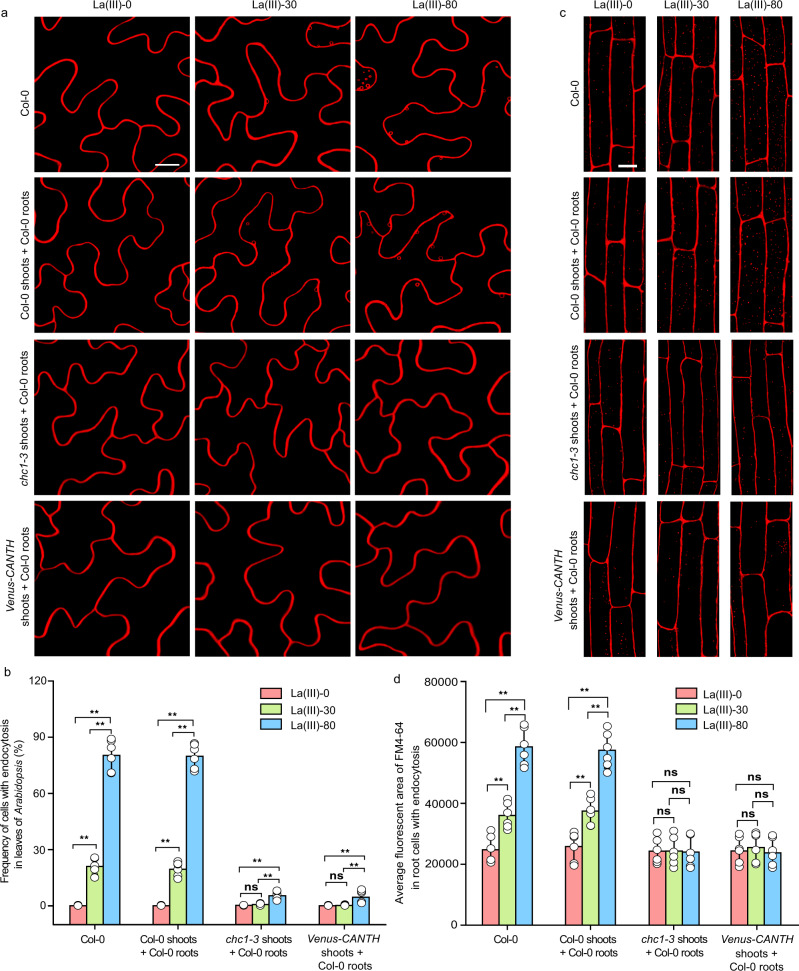
La(III)-induced endocytosis or La(III) internalized by endocytosis in leaves induced systemic endocytosis. **a** The representative CLSM images of Col-0, and the grafted Col-0, *chc1-3*, and DEX-inducible *Venus-CANTH* (shoots from Col-0, *chc1-3*, or DEX-inducible *Venus-CANTH* were grafted onto Col-0 roots) leaf cells treated with 0, 30, or 80 µM La(III) for 12 h and stained with FM4-64. Bar = 5 μm. **b** Quantitative analysis of leaf cells with endocytosis in Col-0, the grafted Col-0, *chc1-3*, and DEX-inducible *Venus-CANTH* (shoots from Col-0, *chc1-3* or DEX-inducible *Venus-CANTH* were grafted onto Col-0 roots) (data come from **a**). **c** The representative CLSM images of Col-0, and the grafted Col-0, *chc1-3*, and DEX-inducible *Venus-CANTH* (shoots from Col-0, *chc1-3*, or DEX-inducible *Venus-CANTH* were grafted onto Col-0 roots). The four kinds of *Arabidopsis* leaves were treated with 0, 30, or 80 µM La(III) for 12 h and then roots were stained with FM4-64. Bar = 10 μm. **d** Quantitative analysis of the average fluorescent area of FM4-64 in Col-0, and the grafted Col-0, *chc1-3*, and DEX-inducible *Venus-CANTH* (shoots from Col-0, *chc1-3*, or DEX-inducible *Venus-CANTH* were grafted onto Col-0 roots) root cells with endocytosis (data come from **c**). In (**a**, **c**), representative images from six independent measurements and three biological replicates (each replicate represents an independently treated plant) per measurement are represented. In (**b**, **d**), values shown are means ± SEM, one-way ANOVA analysis with LSD multiple comparisons test (*n* = 6, **p < 0.01, n.s.: no significance).

The La(III)-induced systemic endocytosis could result from one or more of the following possible mechanisms: (1) aerially applied La(III) directly induces a systemic signal translocated from leaves to roots, (2) La(III)-induced endocytosis in leaf cells or locally internalized La(III) generates a systemic signal transported to roots. To distinguish these possible mechanisms, we used several methods to block La(III)-induced endocytosis in leaves. We first performed grafting experiments to investigate whether blocking La(III)-induced endocytosis in leaves by the *chc1-3* mutation [a loss-of-function mutation in clathrin heavy chain 1 (CHC1)^34^] affects this systemic endocytosis. In these experiments, shoots from the *chc1-3* mutant were grafted onto roots from Col-0 (Fig. 2a–d). In these grafted plants, La(III)-induced endocytosis in both leaf and root epidermal cells was greatly reduced (Fig. 2a–d). We then conducted another grafting experiment, in which shoots from dexamethasone (DEX)-inducible *Venus-CANTH* line were grafted onto roots from Col-0. CANTH is the C-terminal region of the ANTH-containing CME adaptor protein, and is shown to specifically block CME when it is fused with Venus and expressed under DEX-inducible system^15,35^. As expected, DEX-induced expression of Venus-CANTH in leaves (Supplementary Fig. 6) blocked La(III)-induced endocytosis in leaf epidermal cells (Fig. 2a, b). Importantly, epidermal cells of the grafted Col-0 roots exhibited a reduction in La(III)-induced systemic endocytosis (Fig. 2c, d) when the expression of Venus-CANTH was specifically induced in the leaves of grafted plants, compared to the mock-treated control Col-0 and grafted control plants (Col-0 shoots were grafted onto Col-0 roots). Furthermore, treatment of Col-0 leaves with tyrphostin A23 (TyrA23), an inhibitor of CME that functions through inducing cytoplasmic acidification^36^, also drastically decreased La(III)-enhanced systemic endocytosis in Col-0 root cells (Supplementary Fig. 7). Taken together, these results suggest that La(III)-induced endocytosis or La(III) internalized by endocytosis in leaves leads to the production of a systemic signal to induce endocytosis in roots.

We next assessed whether this systemic endocytosis is induced by the internalized La(III) or La(III)-induced endocytosis in leaves. We observed endocytosis in flagellin 22 (flg22)-treated Col-0 because the stimulation with flg22 induced its own internalization via CME^37^. In Col-0 plants whose leaves were treated with flg22, systemic endocytosis was also induced (Supplementary Fig. 8). Therefore, we conclude that La(III)-induced endocytosis in leaves induced systemic endocytosis in roots in *Arabidopsis*.

### La(III)-induced systemic endocytosis requires AtrbohD

**Fig. 3:**
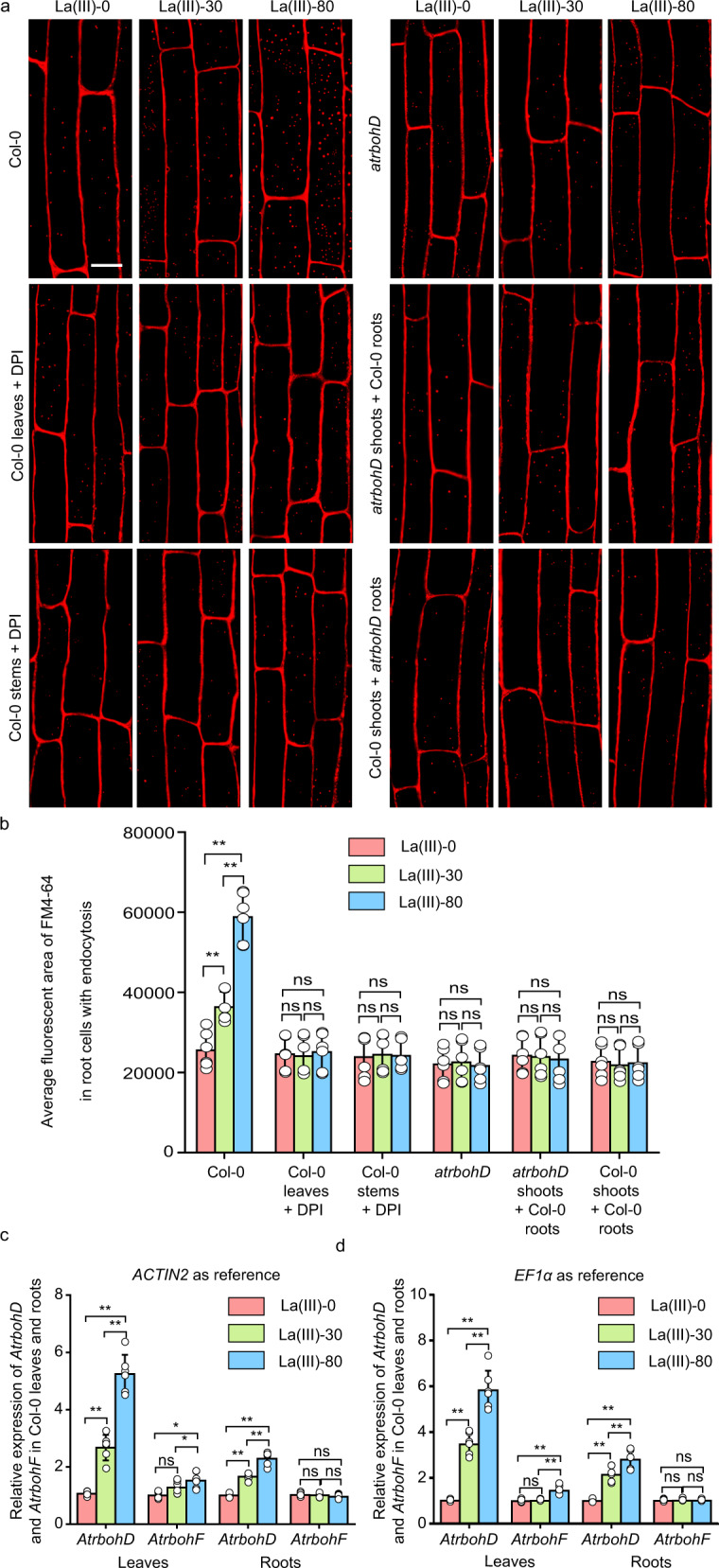
Systemic endocytosis induced by La(III) was dependent on AtrbohD. **a** The representative CLSM images of Col-0, DPI-treated Col-0 (leaves or stems treated with DPI), *atrbohD*, and grafted *atrbohD* (shoots from *atrbohD* were grafted onto Col-0 roots and shoots from Col-0 were grafted onto *atrbohD* roots) root cells. Leaves of all of these *Arabidopsis* were treated with 0, 30, or 80 µM La(III) for 12 h and then roots were stained with FM4-64. Bar = 10 μm. Representative images from six independent measurements and three biological replicates (each replicate represents an independently treated plant) per measurement are represented. **b** Quantitative analysis of the average fluorescent area of FM4-64 in Col-0, Col-0 leaves or stems treated with DPI, *atrbohD*, and grafted *atrbohD* (shoots from *atrbohD* were grafted onto Col-0 roots and shoots from Col-0 were grafted onto *atrbohD* roots) root cells with endocytosis (data come from **a**). Values shown are means ± SEM, one-way ANOVA analysis with LSD multiple comparisons test (*n* = 6, **p < 0.01). **c**, **d** The expression levels of *AtrbohD* and *AtrbohF* transcripts in Col-0 leaves and roots 12 h after treatment of leaves with 0, 30, or 80 μM La(III). The expression levels were quantified by using quantitative RT-PCR. *ACTIN2* (**c**) and *EF1α* (**d**) were as two references. Six independent measurements and three replicates per measurement were conducted. Values shown are means ± SEM, one-way ANOVA analysis with LSD multiple comparisons test (*n* = 6, *p < 0.05, **p < 0.01, n.s.: no significance).

A previous study showed the presence of NADPH oxidase in endocytic vesicles^38^. Furthermore, NADPH oxidase-dependent reactive oxygen species (ROS) have been implicated as systemic signals in plants^39–44^. Therefore, we speculated that NADPH oxidase-mediated ROS might play important roles in transmitting endocytic signals from leaves to roots. To test this hypothesis, we first sprayed diphenyliodonium (DPI), an inhibitor of NADPH oxidase^45^, on the surface of Col-0 leaves and observed endocytosis in root epidermal cells 12 h after La(III) treatment on Col-0 leaves. The DPI treatment greatly reduced La(III)-enhanced endocytosis in root epidermal cells (Fig. 3a, b). Moreover, the application of DPI on stems also greatly reduced endocytosis in root epidermal cells from the same plant whose leaves were treated with La(III) (Fig. 3a, b). These results support the hypothesis that active NADPH oxidase is required for the production of the signal transmitted from leaves to roots to induce systemic endocytosis.

In *Arabidopsis*, AtrbohD and AtrbohF are two key NADPH oxidases responsible for the production of ROS second messengers induced by various primary signals^39,41,46^. To further determine the function of NADPH oxidase in transmitting systemic endocytosis, we first tested the expression levels of *AtrbohD* and *AtrbohF* transcripts in leaves and roots 12 h after treatment of leaves with La(III). We found that in both leaves and roots, the level of *AtrbohD* transcript, but not *AtrbohF* transcript, was greatly increased by treatment of leaves with La(III) (Fig. 3c, d). Importantly, La(III)-induced systemic endocytosis in root epidermal cells of *atrbohD* mutant^46^ was greatly reduced compared to Col-0 when leaves were treated with La(III) for 12 h (Fig. 3a, b). These results suggest that AtrbohD plays a critical role in La(III)-induced systemic endocytosis.

We then tested whether AtrbohD plays a role in generating and/or transmitting the systemic endocytosis signal using reciprocal grafting experiments. The *atrbohD* shoots were grafted onto Col-0 roots, and Col-0 shoots were grafted onto *atrbohD* roots, and their leaves were treated with La(III). In both types of grafted plants, the systemic endocytosis in root epidermal cells was greatly compromised compared to Col-0 (Fig. 3a, b). Thus, AtrbohD is required for triggering systemic endocytosis in roots following the production of a systemic signal from leaves. Taken together, our results suggest that AtrbohD plays a key role in generating and/or transmitting a La(III)-induced long-distance signal from leaves to induce endocytosis in root cells.

### AtrbohD transmits La(III)-induced long-distance endocytic signal through JA

How does AtrbohD transmit the endocytic signal from leaves to roots after La(III) induces endocytosis in leaves? It has been proposed that NADPH oxidase-dependent ROS production mediates long-distance signaling triggered by diverse stimuli^39–44^, and the volatile plant hormone JA has also been implicated in long-distance signaling induced by biotic and abiotic stresses^41,47–49^. To assess whether JA participates in La(III)-triggered long-distance signal transmission to regulate endocytosis in roots, we first determined the levels of JA in leaves and roots 12 h after La(III) treatment on Col-0 leaves using liquid chromatography-MS (LC-MS). La(III) treatment greatly increased the levels of JA in Col-0 leaves and roots, suggesting a link of JA to La(III)-triggered long-distance signal transmission (Supplementary Table 2). The *atrbohD* mutation compromised the increase in the levels of JA in leaves and roots in response to La(III) treatment (Supplementary Table 2). This result suggests a critical role for AtrbohD in the regulation of La(III)-induced JA accumulation and a potential connection between JA and AtrbohD in La(III)-triggered systemic signaling.

**Fig. 4:**
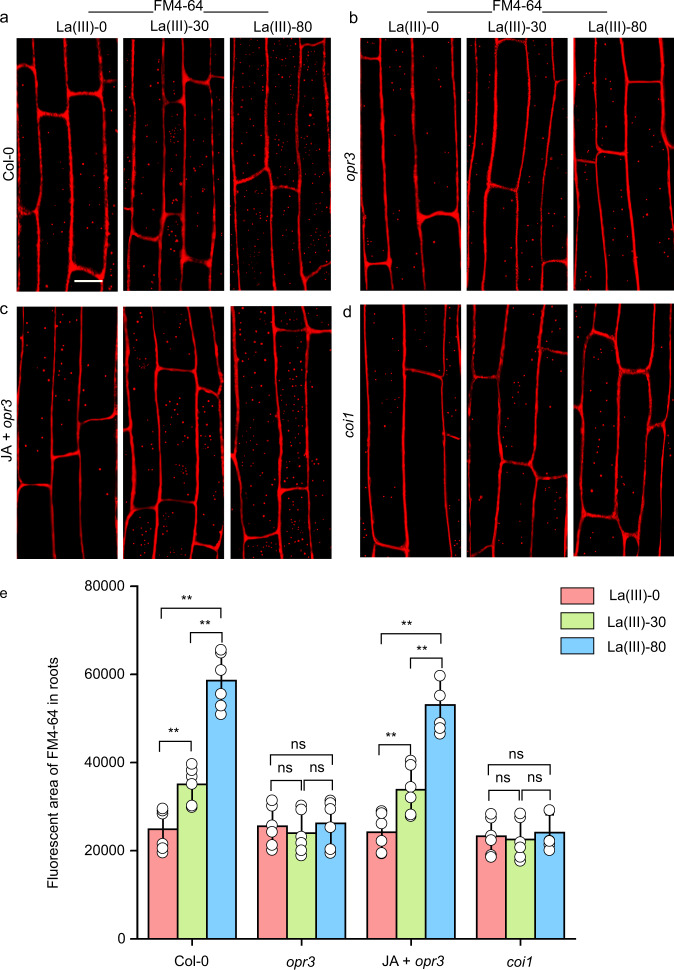
The production of JA changed endocytosis. **a**–**d** Visualization of endocytosis labeled with FM4-64 in Col-0 (**a**), *opr3* (**b**), JA-treated *opr3* (**c**), and *coi1* (**d**) roots 12 h after treatment of leaves with 0, 30, or 80 μM La(III). Bars = 10 μm. Representative images from six independent measurements and three biological replicates (each replicate represents an independently treated plant) per measurement are represented. **e** Quantitative analysis of the fluorescent area of FM4-64 in root cells of Col-0, *opr3*, JA-treated *opr3*, and *coi1* (data come from **a**–**d**). Values shown are means ± SEM. One-way ANOVA analysis with LSD multiple comparisons test (*n* = 6, **p < 0.01, n.s.: no significance).

To test the role of endogenous JA in La(III)-triggered systemic signaling, we used a 12-oxophytodienoate reductase 3 mutant (*opr3*), which is impaired in JA synthesis and is defective in its long-distance signal transduction induced by biotic and abiotic stress^48,49^. The *opr3* mutation greatly compromised systemic endocytosis in root epidermal cells induced by La(III) treatment on leaves (Fig. 4b, e), compared with those in Col-0 root epidermal cells (Fig. 4a, e). Application of JA on *opr3* leaves rescued these defects in La(III)-induced systemic responses (Fig. 4c, e). Furthermore, *OPR3* transcript levels in roots were greatly increased 12 h after treatment of Col-0 leaves with La(III) (Supplementary Fig. 9). Because CORONATINE-INSENSITIVE 1 (COI1) is required for JA signaling^47^, we next asked whether COI1-dependent JA signaling participates in the regulation of La(III)-induced systemic responses. We sprayed La(III) on the surface of *coi1* (impaired in JA signaling^50^) leaves and observed changes in endocytosis in root epidermis cells. In *coi1*, the effects of La(III) on the systemic endocytosis were also greatly compromised compared to Col-0 (Fig. 4d, e). Taken together, these results indicate that La(III)-induced JA biosynthesis and COI1-dependent JA signaling cooperate with AtrbohD in La(III)-triggered systemic signaling to regulate endocytosis.

It was reported that treatment with a low concentration (5 μM) of methyl jasmonate (MeJA) inhibited PIN2 endocytosis in COI1-dependent manner, while a higher concentration (50 μM) reduced PIN2 distribution to the PM^51^. Because La(III) induced JA accumulation in both leaves and roots only to sub-micromolar levels, we next asked whether sub-micromolar JA can activate endocytosis in roots. Treatment of roots with 50 nM JA for 12 h activated endocytosis in root epidermal cells (Supplementary Fig. 10). This is in contrast to the reported inhibition of PIN2 endocytosis by treatment with 5 μM MeJA for 16 h^51^. This result suggests that our observed role of JA in the systemic regulation of endocytosis differs from the inhibition of PIN2 distribution to the PM by high concentrations of MeJA. Thus, the La(III)-triggered systemic regulation of endocytosis by the cooperative action of AtrbohD and JA is unique. How AtrbohD coordinates with JA in the systemic signaling is worthy of future investigation.

### La(III)-induced systemic endocytosis regulates the accumulation of mineral elements in roots and the growth of the whole plant

We next asked whether the systemic endocytosis induced by La(III) is involved in the regulation of La(III)-induced systemic responses of plant growth. To address this question, we first measured the levels of mineral nutrient elements in the roots of Col-0 plants, whose leaves were treated with La(III). ICP-MS analysis showed that 24 h after treatment of leaves with La(III), the levels of major mineral nutrient elements (such as K, Ca, Mg, P, Fe, and Zn) in roots were greatly increased (Supplementary Table 3). Interestingly, treatment with TyrA23 or DPI on leaves negated the accumulation of these mineral nutrient elements induced by aerially applied La(III) (Supplementary Table 3). These data suggest that La(III)-induced systemic endocytosis increased the accumulation of mineral nutrient elements in roots.

To assess whether the La(III)-induced systemic endocytosis may play a role in the regulation of plant growth and development, we measured total leaf areas, primary root length, and lateral root numbers in Col-0 plants, whose leaves were treated with La(III) or co-treated with La(III) and TyrA23, or La(III) and DPI. The results showed that the La(III)-induced effects on leaf expansion, primary root growth, and lateral root formation were all suppressed by co-treatment with either TyrA23 or DPI (Supplementary Table 4). Therefore, aerially applied REEs affect the growth of the whole plant apparently through their regulation of systemic endocytosis.

## Discussion

**Fig. 5:**
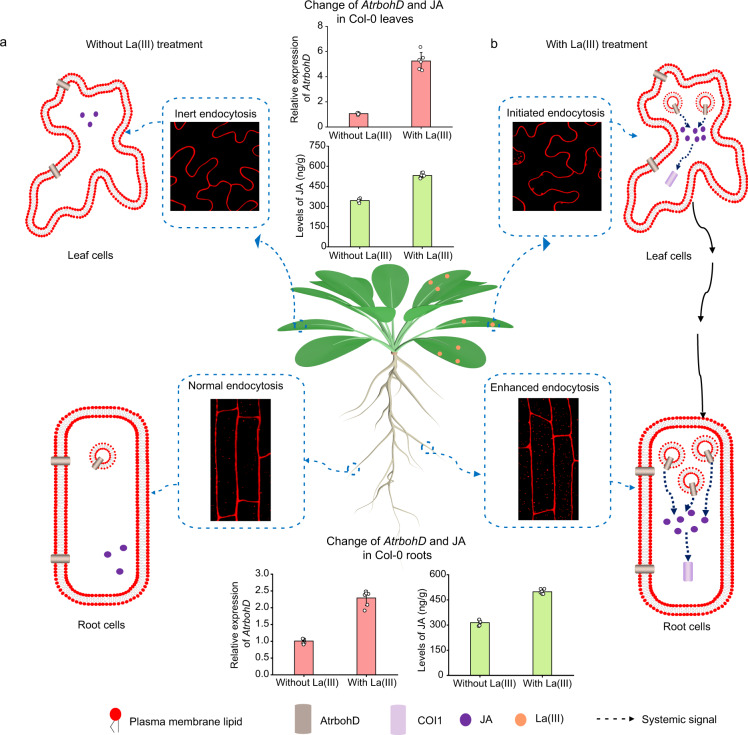
A model for AtrbohD- and JA-dependent systemic endocytosis induced by La(III). **a** Without La(III) treatment, inert endocytosis and normal endocytosis were maintained in leaf and root cells, respectively. Meanwhile, small amounts of JA were produced in leaf and root cells. **b** La(III) treatment on leaves induced CME in leaf cells. AtrbohD promoted the accumulation of JA, and JA further transmitted systemic endocytic signal with the participation of COI1-dependent signaling. This systemic signal induced the enhanced CME in root cells.

In this study, we have provided convincing evidence that La(III)-activated endocytosis in *Arabidopsis* leaves generates an NADPH oxidase-dependent systemic signal to induce endocytosis in roots (Figs. 1–5, Supplementary Figs. 1–10, and Supplementary Tables 1 and 2). This finding begs for many interesting questions. First of all, how widespread is systemic endocytosis? The induction of systemic endocytosis by flg22 supports the common existence of systemic endocytosis (Supplementary Fig. 8). REEs are also internalized in animal cells by CME, and this could provide a strategy for drug delivery in human^17^. It would be of great interest to know whether REEs could also induce systemic endocytosis in animals. Although seventeen REEs have similar physical and chemical properties, their biological effects are not completely identical^4,14^. It would be interesting to see if other REEs, such as Ce(III) or Tb(III)^4,14^, which also initiate endocytosis in leaf cells, could induce systemic endocytosis like La(III). In addition, it has been reported that direct application of REEs on roots could enhance endocytosis of root cells^16,52^. It would also be of importance to know whether the REE-induced systemic endocytosis is bidirectional, i.e., whether there is root-to-shoot systemic endocytosis as well.

Our findings show that the systemic endocytosis induced by La(III) is dependent on AtrbohD-mediated generation of a systemic signal (Figs. 1–5, Supplementary Figs. 1–10, and Supplementary Tables 1 and 2). Our data also suggest AtrbohD plays an important role in the long-distance signal transmission by coordinating with another long-distance signal JA (Figs. 1–5, Supplementary Figs. 1–10, and Supplementary Tables 1–4). We found that AtrbohD regulates the accumulation of JA, and JA is further required for the production of the systemic endocytosis signals induced by La(III). Therefore, these findings support a paradigm for long-distance signal transmission, in which AtrbohD-dependent signal and JA may self-propagate systemic endocytosis signaling over a long distance. How AtrbohD and JA regulate each other for the long-distance transmission of endocytosis signal and how the specificity of systemic signaling is determined will be important future questions. Under stress conditions, ROS is produced via NADPH oxidases and is proposed to propagate between cells as waves to transmit systemic signals over a long distance, which depends on hydrogen peroxide (H_2_O_2_) in ROS^39–43^. It will be interesting to investigate whether H_2_O_2_ or other NADPH oxidase-dependent ROS coordinates with JA in the long-distance signaling that regulates systemic endocytosis.

REEs have been used in agriculture to improve crop productivity for decades, but the underlying mechanisms for REEs’ regulation of plant growth remain unclear^4,13–15,52–55^. Interestingly our results suggest that La(III)-induced systemic endocytosis regulates both leaf expansion and root development (Supplementary Table 4). Furthermore, our findings show that La(III)-induced systemic endocytosis increases the accumulation of mineral nutrient elements in roots (Supplementary Table 3). Therefore, the REE-induced systemic endocytosis likely provides an explanation for REEs’ effect on crop productivity. Nonetheless, elucidating the mechanisms by which the induced systemic endocytosis regulates the accumulation of mineral nutrient elements and plant growth will be an exciting and important future direction.

## Methods

### Reagents

LaCl_3_ (purity >99.99%) was purchased from Aladdin Bio-Chem Technology Co. Ltd (Shanghai, China). FM4-64 was purchased from Thermo Fisher Scientific Co. Ltd (Shanghai, China). DPI, TyrA23, Murashige and Skoog medium (MS), methyl 1-(butylcarbamoyl)-2-benzimidazolecarbamate (Benomyl), 6-benzylaminopurine, indole acetic acid (IAA), and JA were purchased from Sigma-Aldrich Co. Ltd (Shanghai, China). Flg22 was purchased from MedChemExpress Co. Ltd (Shanghai, China). Anti-plant actin mouse monoclonal antibody (A01050) was purchased from Abbkine Scientific Co. Ltd (Wuhan, China). Goat anti-mouse IgG H&L [horseradish peroxidase (HRP)] (ab205719) and HRP anti-GFP antibody (ab6663) were purchased from Abcam Co. Ltd (Shanghai, China). Other chemicals used in this study were analytical reagents and were purchased from Sinopharm Chemical Reagent Co. Ltd. (Shanghai, China).

### Plant materials, growth conditions, and LaCl_3_ treatment

*Arabidopsis thaliana* ecotypes Col-0, *pCLC1::CLC1-GFP* line^28^, DEX-inducible *Venus-CANTH* line^15,35^, and *ap2μ-1* (SALK_083693C)^15^, *chc1-3* (SALK_018351C)^34^, *atrbohD*^46^, *opr3*^48^, and *coi1*^50^ mutants were used in this study. To generate a *pCLC1::CLC1-GFP* line, the CLC1 genomic DNA including its upstream 271 bp was amplified by PCR. The PCR product was cloned into pDONOR to obtain an entry vector. Then, the entry vector was fused into the destination vector pGWB4 vector to obtain *pCLC1::CLC1-GFP*, which was transformed into *A. thaliana* Col-0 by the floral dipping method^56^. A recombinant plasmid for DEX-inducible expression of *Venus-CANTH* was constructed in a derivative of pTA7002, containing gateway cassettes kindly provided by Yuichiro Watanabe from the University of Tokyo^57^. A partial complementary DNA (cDNA) for the AP180 protein (residues 991–1959 of At1g05020) was amplified by PCR. The PCR product was fused into pDONOR to obtain an entry vector, which was fused into the derivative of pTA7002. The resulting plasmid was used to transform *A. thaliana* Col-0 to obtain a DEX-inducible *Venus-CANTH* line. For induction, 5 μM DEX from 30 mM stock solution in ethanol was used. The primers used in generating *CLC1-GFP* and DEX-inducible *Venus-CANTH* lines were listed in Supplementary Table 5.

Seeds were surface sterilized and imbibed for 2 days at 4 °C in the dark, and then plated onto an MS medium (pH 5.7) supplemented with 1% (w/v) sucrose and 1% agar (w/v) at 22 °C. Seedlings were grown in a climate-controlled growth room [22 °C/20 °C (day/night), 100 µmol m^−2^ s^−1^ light intensity and 16/8-h photoperiod (day/night)]. Some five-day-old seedlings with healthy roots were transplanted into the soil and grown in the growth chamber for another 15 days.

All plant treatments, unless specified, were performed as follows: (1) 20-day-old seedlings were sprayed evenly with 5, 30, 55, 80, or 105 μM LaCl_3_ until droplets began to fall and the control seedlings were sprayed evenly with deionized water. Twelve hours, 24 h, or 7 days after spraying, leaves and roots in the same position were sampled for further analyses. (2) Twenty-day-old seedlings were sprayed evenly with 90 or 240 μM NaCl until droplets began to fall and the control seedlings were sprayed evenly with deionized water. Twelve hours later, leaves and roots in the same position were sampled for further analyses. (3) Twenty-day-old seedlings were sprayed evenly with 10 μM DPI or were treated with 10 μM DPI in stems. Then, leaves were sprayed evenly with LaCl_3_ solution (0, 30, or 80 μM) until the droplets began to fall. Twelve hours, 24 h, or 7 days after spraying, leaves and roots in the same position were sampled for further analyses. (4) Twenty-day-old seedlings were sprayed evenly with 30 μM TyrA23^25^. One hour later, leaves were sprayed evenly with LaCl_3_ solution (0, 30, or 80 μM) until droplets began to fall. Twelve hours, 24 h, or 7 days after spraying, leaves and roots in the same position were sampled for further analyses. (5) Twenty-day-old seedlings were sprayed evenly with 0 or 10 μM flg22 until droplets began to fall. Two hours later, leaves and roots in the same position were sampled for further analysis. (6) Twenty-day-old *opr3* was sprayed evenly with 50 nM JA. Sixty minutes later, leaves were sprayed evenly with LaCl_3_ solution (0, 30, or 80 μM) until the droplets began to fall. Twelve hours after spraying, leaves and roots in the same position were sampled for further analyses. (7) Roots of 20-day-old seedlings were treated with 0 or 50 nM JA. Twelve hours after treatment, roots in the same position were sampled for further analyses.

### Grafting of *Arabidopsis* plants

Seedlings to be grafted were germinated on plates containing 0.5× MS with 3 mg L^−1^ Benomyl, 0.04 mg L^−1^ 6-benzylaminopurine, 0.02 mg L^−1^ IAA, and 12 g L^−1^ agar. Treatment with IAA could be found to greatly improve grafting efficiency based on the following factors: enhanced callusing at the grafted union, retardation of shoot growth that maintained contact at the grafted union, and an ~90% reduction in the formation of adventitious roots at the grafted union. Benomyl virtually eliminated fungal contamination. Plates containing the stratified seeds were placed vertically under controlled environmental conditions (16/8-h photoperiod and 25 °C). Five-day-old seedlings were grafted on the plate by the 90° blunt-end technique with a 15° Stab Knife without collars^58^. The grafted seedlings remained on the plate for the next 5 days to allow the formation of the grafted union. Successfully unified seedlings were transplanted directly to the soil (as described above in the “Plant materials, growth conditions, and LaCl_3_ treatment” section).

### Measurements of growth, physiological, and biochemical indices

Growth indices, including the total leaf areas of all leaves, primary root length, and lateral root numbers, were measured. Three independent measurements and ten plants per measurement were conducted. Physiological and biochemical indices, including net photosynthetic rate, chlorophyll content, and thiobarbituric acid-reactive species (TBARS) content, were measured as follows.

The net photosynthetic rate was measured using a portable fluorescence system (GFS-3000, WALZ, Effeltrich, Germany) in a chamber. Light saturation point, temperature, CO_2_ concentration, air flow rate, and relative humidity in the chamber were 800 µmol m^−^^2^ s^−1^, 25 °C, 700 mg m^−3^, 750 µmol s^−1^, and 70%, respectively.

Fresh leaves (~0.1 g) were homogenized with 10 mL extraction solution (80% acetone). Then, samples were centrifuged at 10,000 × *g* for 5 min. The supernatant was collected and its absorbance was measured at 663 and 645 nm (*A*_663_ and *A*_645_) using an ultraviolet–visible spectrophotometer (Evolution^TM^ 60S, Thermo Fisher Scientific, Shanghai, China), respectively. Chlorophyll content was calculated according to Arnon’s equation as shown in the following equation^59^:

1 $${\rm{Chlorophyll}}\;{\rm{content}}\; ({\rm{mg}}\cdot {\mathrm{g}}^{-1}\; {\rm{FW}})=\frac{(8.02{A}_{663}+20.21{A}_{645})\times V}{{\rm{FW}}\times 1000}$$

In this equation, *V* and FW were the volume of the extraction solution (mL) and the fresh weight of leaves (g), respectively.

Fresh leaves (~0.1 g) were homogenized with 1 mL extraction solution (10% trichloroacetic acid). Then, samples were centrifuged at 10,000 × *g* for 10 min. The supernatant was collected and 0.2 mL 0.6% thiobarbituric acid was added to 0.2 mL supernatant to obtain the reaction solution. After reacting in boiling water for 30 min, the reaction solution was cooled and centrifuged at 10,000 × *g* for 10 min. The supernatant was collected and its absorbance was measured at 450, 532, and 600 nm (*A*_450_, *A*_532_, and *A*_600_) using the ultraviolet–visible spectrophotometer, respectively. TBARS content was calculated according to the following equation:

2 $${\rm{TBARS}}\;{\rm{content}}\;({\rm{nmol}}\cdot {\mathrm{g}}^{-1}\; {\rm{FW}})=\frac{[6.45\times ({A}_{532}-{A}_{600})-0.56\times {A}_{450}]\times {V}_{\mathrm{r}}}{{\rm{FW}}\times {V}_{\mathrm{s}}\div{V}_{\mathrm{e}}}$$

In this equation, FW, *V*_r_, *V*_s_, and *V*_e_ were the fresh weight of leaves (g) and the volume of the reaction solution, supernatant in the reaction solution and the extraction solution (mL), respectively.

### Endocytosis observation

Leaves and roots were immersed in 2 μM FM4-64 for 30 min and observed under a CLSM (Leica SP8, Wetzlar, Germany) with a ×63 oil objective. The excitation wavelength for FM4-64 and CLC1-GFP was 514 and 488 nm, respectively. The observation wavelength for FM4-64 and CLC1-GFP was 650 and 515 nm, respectively. Moreover, the dynamic process of endocytosis was observed using spinning disc confocal microscopy with a CSU-X1 spinning disc head (Yokogawa, Tokyo, Japan) equipped with a CFI Apo TIRF 1003 NA1.49 oil immersion objective and an Evolve EMCCD camera.

For observing the morphology of endocytosis, leaves (2–3 mm in length and width) were fixed in the solution containing 2% paraformaldehyde and 1% glutaraldehyde solution and postfixed with 1% osmic acid. After fixation and dehydration, samples were embedded in epoxy resin. Following epoxy resin polymerization, thin sections were prepared with a Reichert-Jung Ultracut E ultramicrotome (Vienna, Austria). Then, the thin sections were observed under a TEM (HITACH H-7650, Tokyo, Japan).

### Western blotting assay

To investigate Venus-CANTH expression in the DEX-inducible *Venus-CANTH* line, western blotting assay was performed. Total proteins were extracted from leaves by using 1 mL of Protein Extraction Buffer [50 mM Tris-HCl (pH 7.4), 5 mM EDTA, 150 mM NaCl, 1% Triton X-100], 10 μL of 100× phenylmethanesulfonyl fluoride, and 10 μL of 100× protease inhibitor cocktail. Then, the samples were subjected to sodium dodecyl sulfate-polyacrylamide gel electrophoresis and analyzed by western blotting. Anti-plant actin mouse monoclonal antibody (1:2000 dilution) and monoclonal goat anti-mouse IgG H&L (HRP) (1:2000 dilution) were used for detecting actin. HRP anti-GFP antibody (1:2000 dilution) was used for detecting Venus-CANTH.

### Quantitative real-time PCR (qRT-PCR) analysis

To investigate the expression levels of *AtrbohD*, *AtrbohF*, and *OPR3*, qRT-PCR analysis was performed. Total RNA was isolated using an RNA purification kit. The total RNA was reverse transcribed using the PrimeScript RT Reagent Kit with gDNA Eraser. For qRT-PCR analysis, the primers of *AtrbohD*, *AtrbohF*, and *OPR3* were used to determine their expression levels, and these primers were listed in Supplementary Table 5. *ACTIN2* and *EF1α* were used as references.

### Osmotic pressure analysis

Fresh leaves or roots (0.05 g) were frozen with liquid nitrogen and squeezed in a syringe as soon as the frozen leaves melt. The liquid obtained from squeeze was centrifuged at 11,300 × *g* for 5 min. Then, the supernatant was diluted with deionized water and centrifuged to remove air bubbles. Osmotic pressure was measured using a freezing point osmometer (OSMOMAT 030, Berlin, Germany).

### JA quantification

Fresh leaves or roots (0.2 g) were homogenized in an ice bath with 0.5 mL extraction buffer [isopropanol:water:HCl = 2:1:0.005 (v/v/v)] and agitated for 30 min at 4 °C. Then, samples were dissolved in 200 μL of 80% methanol after extraction with dichloromethane and solvent evaporation, and analyzed using LC (Agilent 1290 Infinity LC, Santa Clara, USA)-MS (Agilent 6460 QQQ MS, Santa Clara, USA). JA was separated on an Eclipse XD8-C18 HPLC column (3 μm, 150 × 2.00 mm^2^, Agilent, Santa Clara, USA) using a gradient of 0.1% formic acid in water (solvent A) and 0.1% formic acid in acetonitrile (solvent B) at flow rate of 600 μL min^−1^.

### Metal element and P level analysis

Twenty-four hours after spraying Col-0 seedlings with 0, 30, or 80 μM LaCl_3_, the roots were washed five times with deionized water and dried at 65 °C until they were a constant weight. After grinding, the mixture was digested in concentrated nitric acid and H_2_O_2_ in a multi-wave sample preparation system (Multiwave 3000, Anton Paar, Austria), in which 5 mL nitric acid and 0.5 mL H_2_O_2_ were added in a Teflon perfluoroalkoxy digestion vessel. After 1 h digestion, the digestion solution was diluted with micro-filtered deionized water for five times. The supernatant was centrifuged at 4000 r.p.m. for 5 min, and the levels of La and mineral nutrient elements (K, Ca, Mg, P, Fe, and Zn) were determined by XSERIES 2 ICP-MS (Thermo Fisher Scientific, Bremen, Germany). Moreover, the levels of mineral nutrient elements (K, Ca, Mg, P, Fe, and Zn) in La(III) and TyrA23 co-treated Col-0, or La(III) and DPI co-treated Col-0 were also determined according to this method.

### Statistical analyses

All experiments, unless specified, were conducted for six independent measurements (six times), and each treatment group in each measurement contained three replicates. The results are expressed as the mean ± SEM. One-way analysis of variance with least significant difference multiple comparisons test (*p* < 0.05 or *p* < 0.01) was used for the analysis of significant differences.

### Reporting summary

Further information on research design is available in the Nature Research Reporting Summary linked to this article.

## Supplementary information

Supplementary Information

Descriptions of Additional Supplementary Files

Supplementary Movie 1

Reporting Summary

## Data Availability

All data included in the manuscript and/or Supplemental materials are available from the corresponding authors upon reasonable request. Source data are provided with this paper.

## Source data

Source Data
