# Supplementary material for: Lanthanum(III) triggers AtrbohD- and jasmonic acid-dependent systemic endocytosis in plants
Source: Nat Commun. 2021 Jul 15;12:4327. doi: 10.1038/s41467-021-24379-z (PMC8282819; doi:10.1038/s41467-021-24379-z)
Supplement: Supplementary file 1 — Supplementary Information [file 41467_2021_24379_MOESM1_ESM.pdf]

Supplementary information

Lanthanum(III) triggers AtrbohD- and jasmonic acid-dependent systemic endocytosis in plants

Mengzhu Cheng[1,8], Lihong Wang[1,2,8], Qing Zhou[2], Daiyin Chao[3], Shingo Nagawa[4,5], Ding He[2], Jiazhi Zhang[2], Hui Li[5,6], Li Tan[5], Zhenhong Gu[5], Xiaohua Huang[1]✉ and Zhenbiao Yang[7]✉

[1]National and Local Joint Engineering Research Center of Biomedical Functional Materials, Jiangsu Collaborative Innovation Centre of Biomedical Functional Materials, School of Chemistry and Materials Science, Nanjing Normal University, Nanjing, China. [2]State Key Laboratory of Food Science and Technology, Jiangnan University, Wuxi, China. [3]National Key Laboratory of Plant Molecular Genetics, Institute of Plant Physiology and Ecology, Shanghai Institutes for Biological Sciences, Chinese Academy of Sciences, Shanghai, China. [4]Fujian Agriculture and Forestry University-University of California, Riverside, Joint Center for Horticultural Biology and Metabolomics, Haixia Institute of Science and Technology, Fujian Agriculture and Forestry University, Fuzhou, China. [5]Shanghai Center for Plant Stress Biology, Shanghai Institute of Biological Sciences, Chinese Academy of Sciences, Shanghai, China. [6]School of Life Sciences, East China Normal University, Shanghai, China. [7]Center for Plant Cell Biology, Institute of Integrative Genome Biology, and Department of Botany and Plant Sciences, University of California, Riverside, CA, USA. [8]These authors contributed equally: Mengzhu Cheng, Lihong Wang. ✉e-mail: huangxiaohuanjnu@yahoo.com; yang@ucr.edu

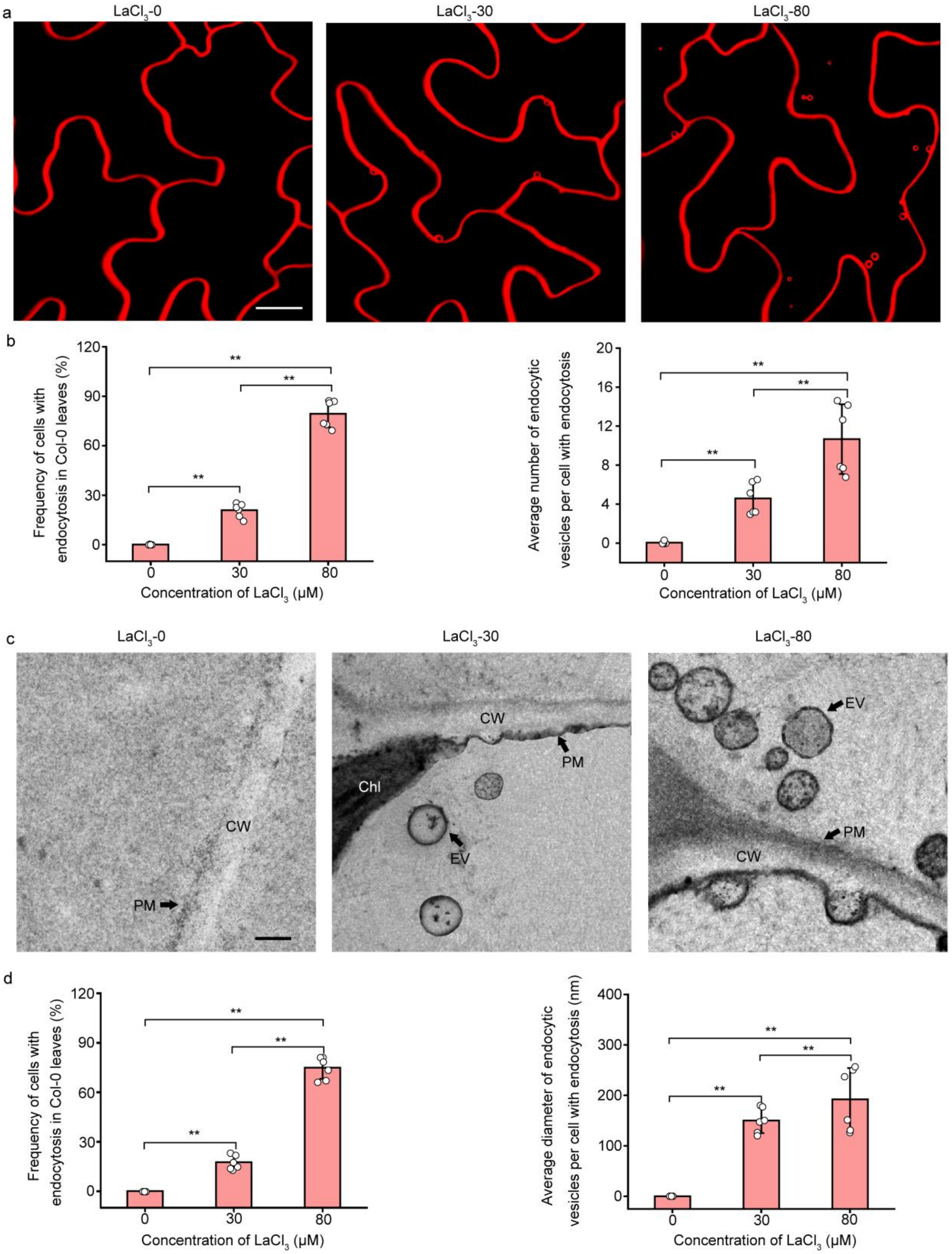

Supplementary Fig. 1. $LaCl_3$ induced endocytosis in leaf cells. a The representative CLSM images of Col-0 leaf epidermal cells treated with 0, 30 or 80 μM $LaCl_3$ for 12 h and stained with FM4-64.

Bar=5 μm. **b** Quantitative analysis of the frequency of Col-0 leaf epidermal cells with endocytosis and the average number of endocytic vesicles in these cells (data come from **a**). **c** The representative TEM images of Col-0 leaf cells treated with 0, 30 or 80 μM $LaCl_3$ for 12 h. Bar=200 nm. CW: cell wall; PM: plasma membrane; Chl: chloroplast; EV: endocytic vesicle. **d** The frequency of Col-0 leaf cells with endocytosis and the average diameter of endocytic vesicles in these cells (data come from **c**). Representative images from six independent measurements and three biological replicates (each replicate represents an independently treated plant) per measurement are represented in **a** and **c**. In **b** and **d**, values shown are means ± SEM, one-way ANOVA analysis with LSD multiple comparisons test (n=6, ** p<0.01).

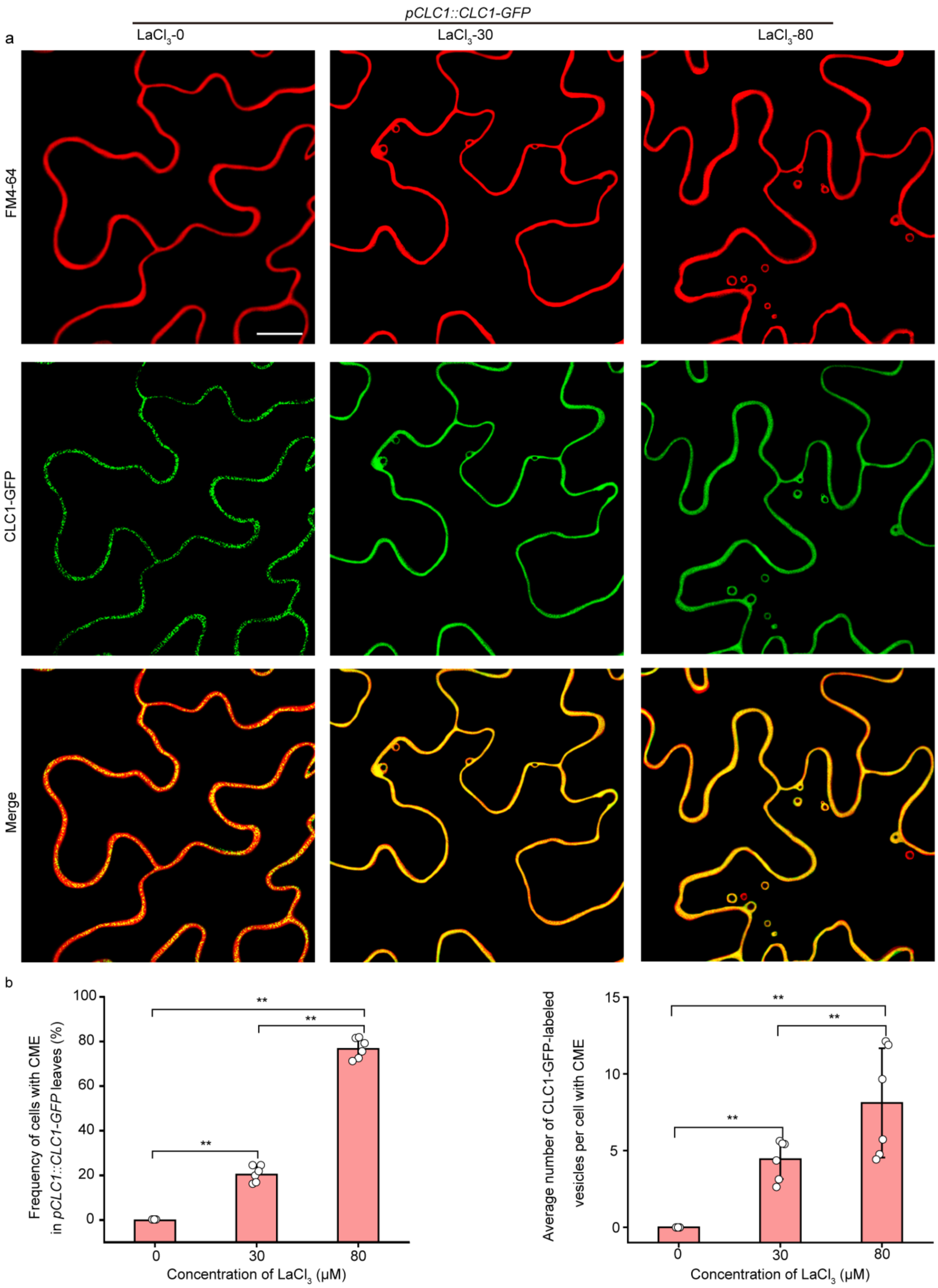

Supplementary Fig. 2. $LaCl_3$ induced CME in leaf cells. a Representative CLSM images of

pCLC1::CLC1-GFP leaf cells treated with 0, 30 or 80 $LaCl_3$ for 12 h and stained with FM4-64. Bar=5 μm. Representative images from six independent measurements and three biological replicates (each replicate represents an independently treated plant) per measurement are represented. A time-lapse imaging of CLC1-GFP endocytic events is shown in **Supplementary Movie 1**. **b** The frequency of *pCLC1::CLC1-GFP* leaf cells with CME and the average number of CLC1-GFP-labeled vesicles in these cells (data come from **a**). Values shown are means ± SEM, one-way ANOVA analysis with LSD multiple comparisons test (n=6, ** p<0.01).

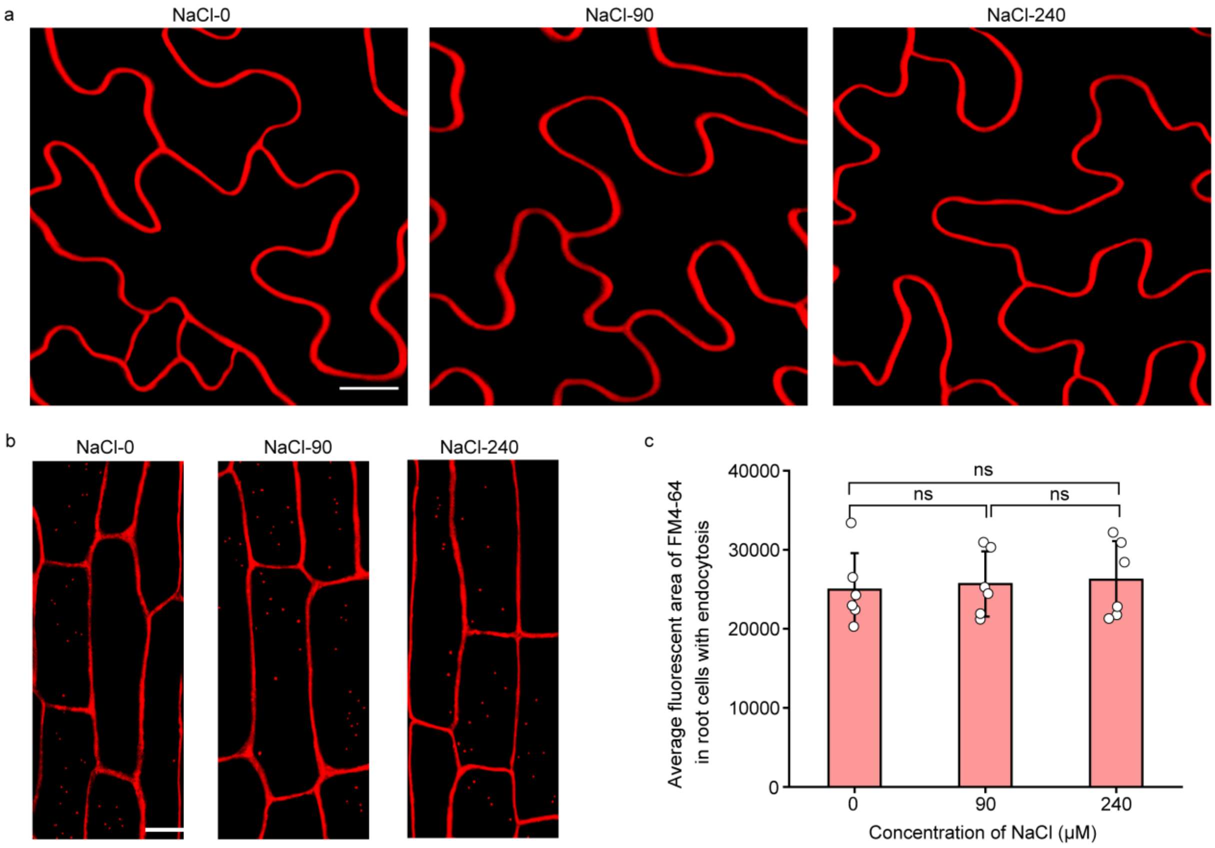

Supplementary Fig. 3. Endocytosis in Col-0 leaves and roots after treatment of leaves with 0, 90 or 240 μM NaCl. a, **b** The representative CLSM images of Col-0 leaves (**a**) and roots (**b**) after treatment of leaves with 0, 90 or 240 μM NaCl for 12 h and staining with FM4-64. The molar concentration of Cl^- in NaCl equals to that in $LaCl_3$. Bar=5 and 10 μm in **a** and **b**, respectively. **c**, Quantitative analysis of the average fluorescent area of FM4-64 in root cells of NaCl-treated Col-0 (data come from **b**). The fluorescent area of FM4-64 was quantified using ImageJ software. Six independent measurements and three replicates (each replicate represents an independently treated plant) per measurement were conducted. Values shown are means ± SEM, one-way ANOVA analysis with LSD multiple comparisons test (*n*=6, ns: no significance).

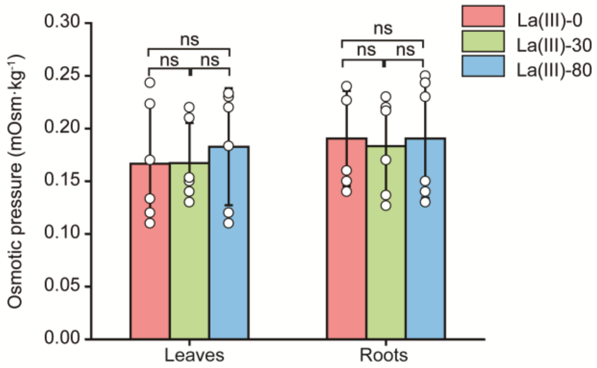

Supplementary Fig. 4. Osmotic pressures of leaves and roots after treatment of leaves with 0, 30 or 80 μM $LaCl_3$ for 12 h. Osmotic pressures of leaves and roots were quantified by using freezing point osmotic pressure gauge. Six independent measurements and three replicates per measurement were conducted. Values shown are means ± SEM, one-way ANOVA analysis with LSD multiple comparisons test (*n*=6, ns: no significance).

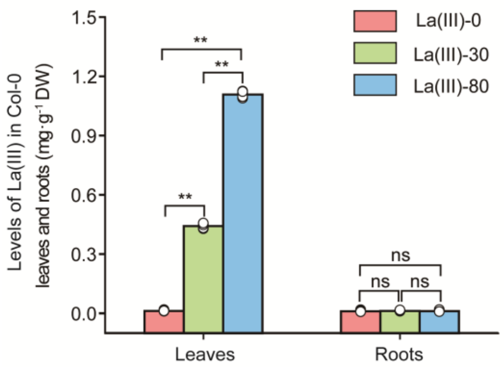

Supplementary Fig. 5. The levels of La(III) in Col-0 leaves and roots 12 h after treatment of leaves with 0, 30 or 80 μM La(III). The levels of La(III) in leaves and roots were quantified by using ICP-MS. Six independent measurements and three replicates per measurement were conducted. Values shown are means ± SEM, one-way ANOVA analysis with LSD multiple comparisons test (n=6, ** p<0.01, ns: no significance).

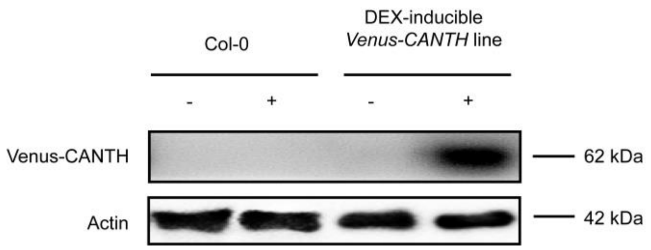

Supplementary Fig. 6. DEX treatment induced Venus-CANTH expression in DEX-inducible *Venus-CANTH* line. Protein extracts from Col-0 and DEX-inducible *Venus-CANTH* line were subjected to protein gel blotting, and the Venus-CANTH expression were analyzed.

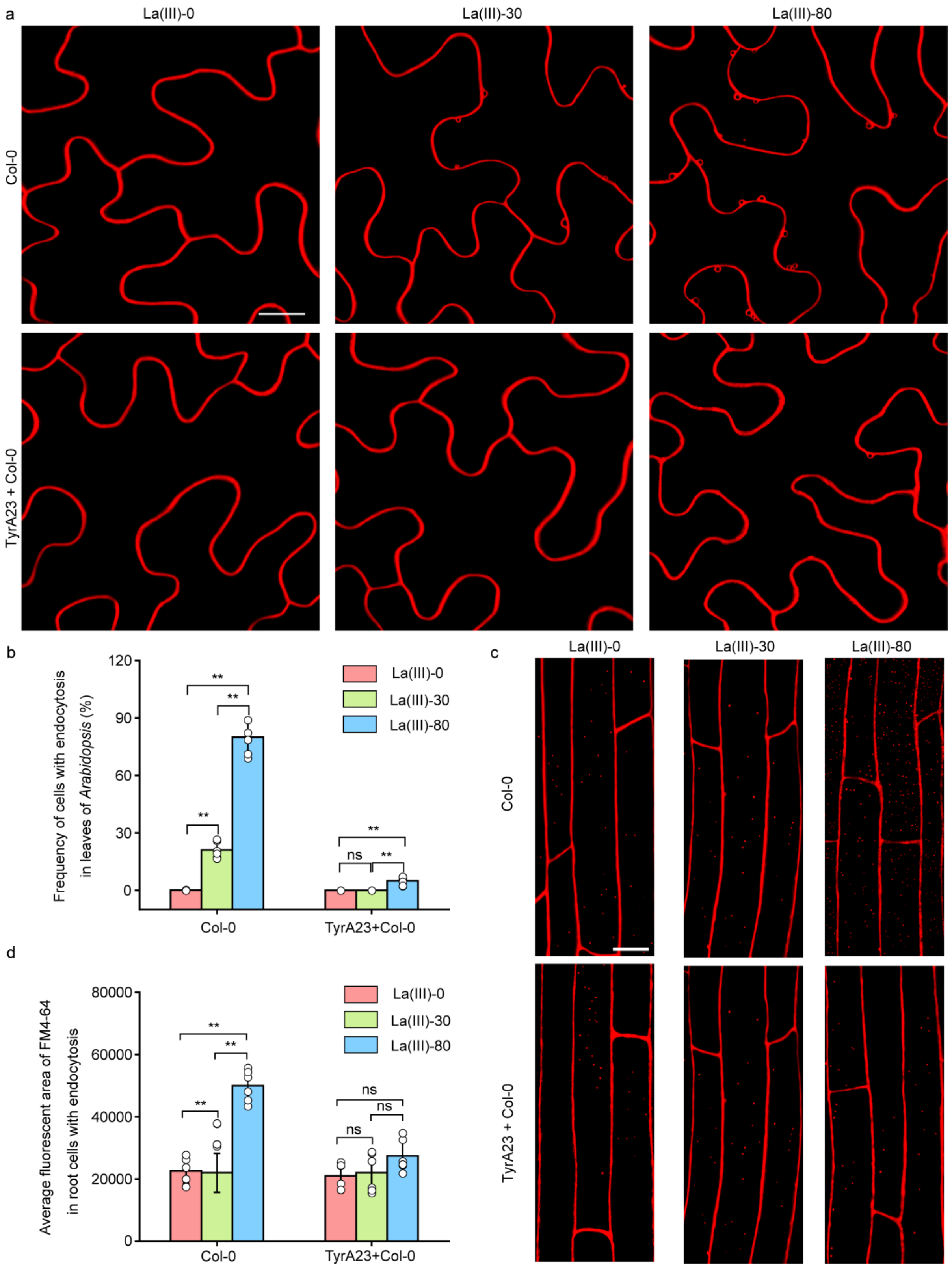

Supplementary Fig. 7. Endocytosis in Col-0 leaves and roots after treatment of leaves with TyrA23 and La(III). a The representative CLSM images of Col-0 leaves after treatment of leaves

with 0, 30 or 80 μM La(III) for 12 h and staining with FM4-64. Leaves were treated without or with TyrA23 before La(III) treatment. Bar=5 μm. **b** Quantitative analysis of leaf cells with endocytosis in Col-0 and TyrA23-treated Col-0 (data come from **a**). **c** The representative CLSM images of Col-0 roots after treatment of leaves with 0, 30 or 80 μM La(III) for 12 h and staining with FM4-64. Leaves were treated without or with TyrA23 before La(III) treatment. Bar=10 μm. **d** Quantitative analysis of the average fluorescent area of FM4-64 in Col-0 and TyrA23-treated Col-0 root cells with endocytosis (data come from **c**). Representative images from six independent measurements and three biological replicates (each replicate represents an independently treated plant) per measurement are represented in **a** and **c**. In **b** and **d**, values shown are means ± SEM, one-way ANOVA analysis with LSD multiple comparisons test (n=6, ** p<0.01).

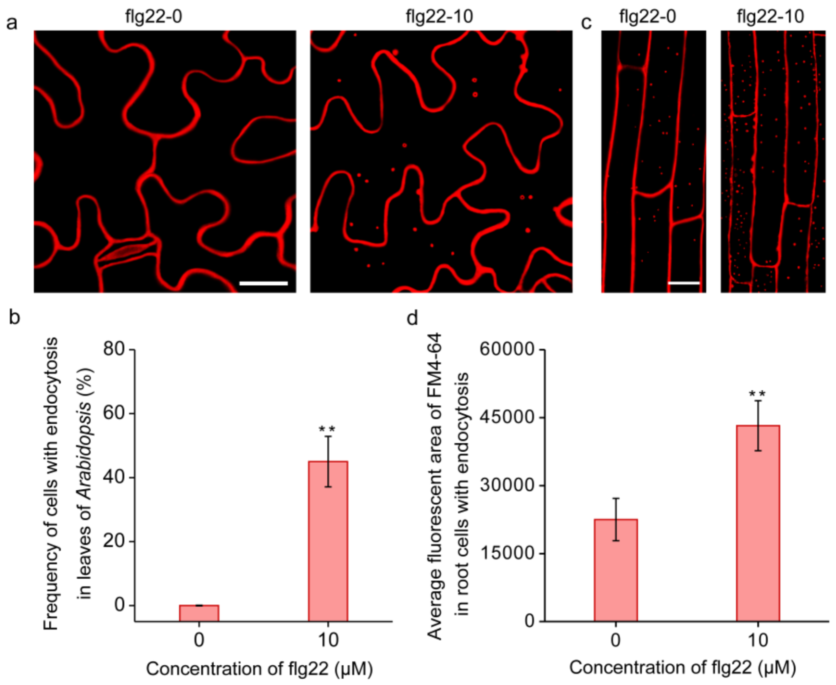

Supplementary Fig. 8. Endocytosis in Col-0 leaves and roots after treatment of leaves with 0 or 10 μM flg22. a The representative CLSM images of Col-0 leaves after treatment of leaves with 0 or 10 μM flg22 for 2 h and staining with FM4-64. Bar=5 μm. **b** Quantitative analysis of leaf cells with endocytosis in Col-0 (data come from **a**). **c** The representative CLSM images of Col-0 roots after treatment of leaves with 0 or 10 μM flg22 for 2 h and staining with FM4-64. Bar=10 μm. **d** Quantitative analysis of the average fluorescent area of FM4-64 in Col-0 root cells with endocytosis (data come from **c**). Representative images from six independent measurements and three biological replicates (each replicate represents an independently treated plant) per measurement are represented in **a** and **c**. In **b** and **d**, values shown are means ± SEM, one-way ANOVA analysis with LSD multiple comparisons test (n=6, ** p<0.01).

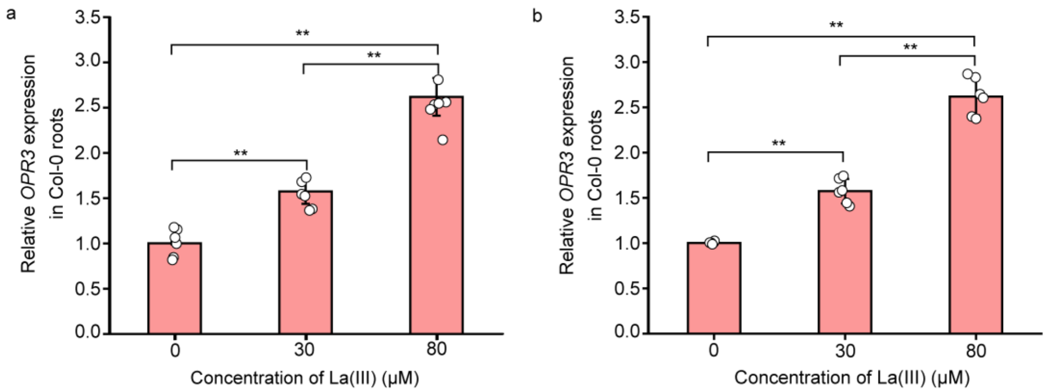

Supplementary Fig. 9. The expression levels of *OPR3* transcripts in Col-0 roots 12 h after treatment of leaves with 0, 30 or 80 μM La(III). The expression levels were quantified by using quantitative qRT-PCR. Six independent measurements and three replicates per measurement were conducted. Values shown are means ± SEM, one-way ANOVA analysis with LSD multiple comparisons test (n=6, ** p<0.01). **a** *ACTIN2* was as reference. **b** *EF1α* was as reference.

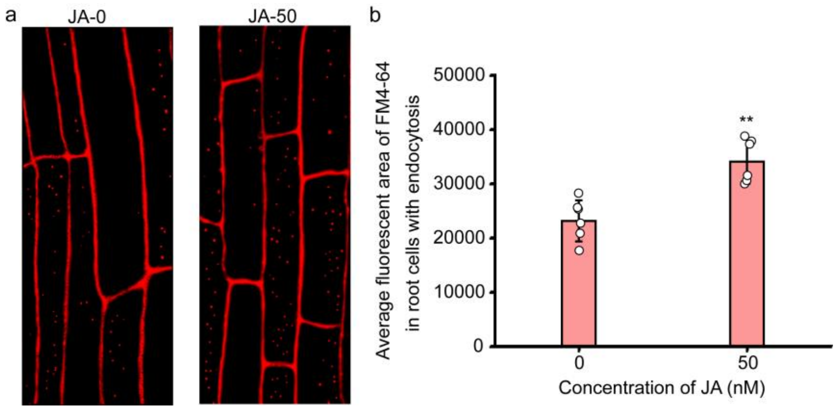

Supplementary Fig. 10. Endocytosis in Col-0 roots after treatment of roots with 0 or 50 nM JA. a The representative CLSM images of Col-0 roots after treatment of roots with 0 or 50 nM JA for 12 h and staining with FM4-64. Bar=10 µm. **b** Quantitative analysis of average fluorescent area of FM4-64 in root cells of JA-treated Col-0 (data come from **a**). The fluorescent area of FM4-64 was quantified using ImageJ software. Six independent measurements and three replicates (each replicate represents an independently treated plant) per measurement were conducted. Values shown are means ± SEM, one-way ANOVA analysis with LSD multiple comparisons test (n=6, ** p<0.01).

Supplementary Table 1. **The growth, physiological and biochemical indices in Col-0 after treatment of leaves with 0, 5, 30, 55, 80 or 105 µM $LaCl_3$ ‡.**

	Concentration of $LaCl_3$ (µM)					
	0	5	30	55	80	105
Total leaf areas* (cm^2)	8.4 ± 0.6^{b}	8.7 ± 0.6^{b}	10.9 ± 0.7^{a}	9.8 ± 0.5^{a}	7.1 ± 0.6^{c}	6.5 ± 0.8^{c}
Primary root length* (cm)	8.0 ± 0.4^{c}	8.2 ± 0.5^{bc}	9.9 ± 0.6^{a}	9.0 ± 0.5^{b}	6.2 ± 0.4^{d}	6.0 ± 0.4^{d}
Lateral root numbers*	23.8 ± 3.0^{cd}	24.5 ± 3.3^{bc}	29.2 ± 2.5^{ab}	27.5 ± 2.8^{abc}	19.2 ± 2^{de}	17.0 ± 2.5^{e}
Net photosynthetic rate† (µmol/m^2/s)	2.05 ± 0.08^{c}	2.10 ± 0.05^{c}	3.58 ± 0.10^{a}	3.31 ± 0.13^{b}	1.43 ± 0.06^{d}	1.21 ± 0.10^{e}
Chlorophyll content† (mg/g FW)	1.54 ± 0.11^{b}	1.57 ± 0.15^{b}	1.76 ± 0.10^{a}	1.68 ± 0.11^{a}	1.38 ± 0.14^{c}	1.29 ± 0.15^{c}
TBARS content† (nmol/g FW)	1.83 ± 0.03^{d}	1.80 ± 0.05^{d}	1.65 ± 0.07^{e}	1.96 ± 0.06^{c}	2.68 ± 0.05^{b}	2.99 ± 0.07^{a}

* The growth indices including total leaf areas, primary root length and lateral root numbers were measured after treatment of leaves with 0, 5, 30, 55, 80 or 105 µM $LaCl_3$ for 7 d. Three independent measurements and ten plants per measurement were conducted.

† The physiological and biochemical indices including net photosynthetic rate, chlorophyll content and TBARS content were measured after treatment of leaves with 0, 5, 30, 55, 80 or 105 µM $LaCl_3$ for 24 h. Six independent measurements and three replicates per measurement were conducted.

‡ Values shown are means ± SEM, one-way ANOVA analysis with LSD multiple comparisons test (For growth indices, n=3; for physiological and biochemical indices, n=6). Significant differences at p<0.05 were denoted with different letters.

Supplementary Table 2. The levels of JA in Col-0 and *atrbohD* leaves, and Col-0 roots 12 h after treatment of leaves with La(III) [*, †]

Plants	Organs	Concentration of La(III) (μM)	Levels of JA (ng/g)
Col-0	Leaves	0	344.7±15.3[c]
		30	434.3±22.1[b]
		80	531.8±18.8[a]
	Roots	0	315.2±16.8[c]
		30	381.2±19.3[b]
		80	499.2±13.8[a]
atrbohD	Leaves	0	388.3±24.0[b]
		30	417.3±25.8[b]
		80	454.7±19.4[a]
	Roots	0	350.2±25.7[a]
		30	355.7±26.8[a]
		80	356.8±26.6[a]

[*] Col-0 and *atrbohD* leaves were treated with 0, 30, or 80 μM La(III). The levels of JA in leaves and roots were quantified by using LC-MS. Six independent measurements and three replicates per measurement were conducted. Values shown are means ± SEM, one-way ANOVA analysis with LSD multiple comparisons test (n=6). Significant differences at p<0.05 were denoted with different letters.

[†] Representative LC-MS images can be found in Supplementary Fig. 2 in Source data.

Supplementary Table 3. The levels of nutrient elements in roots of Col-0, TyrA23-treated Col-0, and DPI-treated Col-0 24 h after treatment of leaves with La(III) *

Time	Plants	Concentration of La(III) (μM)	Levels of K (mg/g $_{DW}$)	Levels of Ca (mg/g $_{DW}$)	Levels of Mg (mg/g $_{DW}$)	Levels of P (mg/g $_{DW}$)	Levels of Fe (μg/g $_{DW}$)	Levels of Zn (μg/g $_{DW}$)
24 h	Col-0	0	15.32±0.65^{c}	6.05±0.43^{c}	1.91±0.14^{b}	4.78±0.32^{c}	513.06±20.15^{c}	402.22±15.27^{c}
		30	21.69±0.83^{a}	10.32±0.28^{b}	2.52±0.12^{a}	6.58±0.53^{b}	595.29±18.57^{b}	447.36±13.82^{b}
		80	18.51±1.02^{b}	16.85±0.35^{a}	2.63±0.09^{a}	9.32±0.44^{a}	706.67±31.56^{a}	531.50±20.89^{a}
	TyrA23-treated Col-0	0	16.05±0.57^{c}	6.24±0.51^{b}	1.84±0.11^{b}	4.83±0.52^{a}	518.82±22.38^{b}	408.41±14.55^{b}
		30	15.67±0.22^{b}	6.18±0.16^{b}	1.98±0.13^{b}	5.01±0.38^{a}	548.18±25.33^{b}	422.3±14.73^{b}
		80	18.11±0.18^{a}	7.28±0.25^{a}	2.13±0.18^{a}	5.43±0.28^{a}	610.87±35.61^{a}	450.43±15.05^{a}
	DPI-treated Col-0	0	13.53±0.31^{b}	5.32±0.34^{b}	1.63±0.06^{a}	4.06±0.32^{a}	433.52±35.13^{a}	371.36±16.35^{a}
		30	13.85±1.02^{b}	5.56±0.31^{b}	1.66±0.16^{a}	4.23±0.4^{a}	456.73±33.45^{a}	389.23±15.5^{a}
		80	14.97±0.85^{a}	7.92±0.15^{a}	1.73±0.05^{a}	4.31±0.39^{a}	458.55±51.16^{a}	380.54±17.13^{a}

* Leaves from all plants were treated with indicated concentrations of La(III) for 24 h and roots were harvested for analysis. The levels of nutrient elements in roots were quantified by using ICP-MS. Six independent measurements and three replicates per measurement were conducted. Values shown are means ± SEM, one-way ANOVA analysis with LSD multiple comparisons test (n=6). Significant differences at p<0.05 were denoted with different letters.

Supplementary Table 4. Total leaf areas, primary root length and lateral root numbers of Col-0, TyrA23-treated Col-0, and DPI-treated Col-0 7 d after treatment of leaves with La(III) [*]

Plants	Concentration of La(III) (μM)	Total leaf areas (cm^2)	Primary root length (cm)	Lateral root numbers
Col-0	0	8.2±0.5^{c}	8.1±0.5^{b}	23.7±3.2^{b}
	30	10.8±0.6^{a}	9.8±0.5^{a}	29.0±2.6^{a}
	80	7.1±0.6^{b}	6.2±0.4^{c}	19.2±2.0^{b}
TyrA23-treated Col-0	0	7.9±0.8^{a}	7.7±0.5^{a}	21.3±3.5^{a}
	30	8.1±0.5^{a}	7.9±0.4^{a}	22.5±2.6^{a}
	80	7.7±0.8^{a}	7.6±0.7^{a}	21.5±3.0^{a}
DPI-treated Col-0	0	8.1±0.6^{a}	8.0±0.4^{a}	23.0±3.7^{a}
	30	8.4±0.7^{a}	7.8±0.4^{a}	22.5±3.5^{a}
	80	8.0±0.6^{a}	7.8±0.5^{a}	22.8±3.5^{a}

[*] Leaves from all plants were treated with indicated concentrations of La(III) for 7 d, and then leaves or roots were harvested for analysis. Three independent measurements and ten plants per measurement were conducted. Values shown are means ± SEM, one-way ANOVA analysis with LSD multiple comparisons test (*n*=3). Significant differences at $p<0.05$ were denoted with different letters.

Supplementary Table 5. Primer sequences used in molecular clone and qRT-PCR analysis

Primer sequences used in molecular clone		
Transgenetic line	Oligo name	Primer Fw Sequence (5´-3´)
pCLC1::CLC1-GFP	CLC1-207F	GGGGACAAGTTTGTACAAAAAAGCAGGCTGCTGTCGTCTCTTTCCGTTAAAG
	CLC1-207R	GGGGACCACTTTGTACAAGAAAGCTGGGTCCTCCGCCTTGGTTCCCTCGGC
DEX-inducible *Venus-CANTH*	CANTH-F	TCGTTTCCAGCGAGCTCAG
	CANTH-R	ACTCAAGTGCTTGGCTATGA

Primer sequences used in qRT-PCR analysis		
Gene name/AGI code	Primer Fw Sequence (5´-3´)	Primer Rev Sequence (5´-3´)
ACTIN2/AT3G18780	GTGGATTCCAGCAGCTTCCAT	GCTGAGAGATTCAGATGCCCA
EF1α/AT5G60390	ACGCTCTTCTTGCTTTCACC	GAGATTGGCACAAATGGGAT
AtrbohD/AT5G47910	TCCACGCACTCAAAGGTCTC	CGTTGGAATCAGCGGAGAGT
AtrbohF/AT1G64060	ACGTTTAGCTCCACACGTTCT	TGCTTCTATCAAACCCCCTTGA
OPR3/AT2G06050	ATCTCGGAAAACAGGTGGCG	CGGAAGCTTCTAAAGCCCGA
