# Supplementary material for: Lanthanum(III) triggers AtrbohD- and jasmonic acid-dependent systemic endocytosis in plants
Source: Nat Commun. 2021 Jul 15;12:4327. doi: 10.1038/s41467-021-24379-z (PMC8282819; doi:10.1038/s41467-021-24379-z)
Supplement: Supplementary file 2 — Descriptions of Additional Supplementary Files [file 41467_2021_24379_MOESM2_ESM.pdf]

Descriptions of Additional Supplementary Files

Supplementary Movie 1

Description: Real-time dynamic of CME in *pCLC1::CLC1-GFP* leaf cells treated with 0 or 30 μM $LaCl_3$, related to Supplementary Fig. 2. $LaCl_3$-induced CME was visualized with FM4-64 and GFP-tagged CLC1 using a spinning disc confocal microscopy with a CSU-X1 spinning disc head. The time-lapse images were captured with an interval of 1 s, and then be integrated into a movie. No endocytic vesicle emerged from the plasma membrane (PM) in the control, and clathrin-mediated endocytic vesicles formed from pinching off the PM after $LaCl_3$ treatments. These results suggest that $LaCl_3$ induces CME in leaf cells.
