# Supplementary material for: Lanthanum(III) triggers AtrbohD- and jasmonic acid-dependent systemic endocytosis in plants
Source: Nat Commun. 2021 Jul 15;12:4327. doi: 10.1038/s41467-021-24379-z (PMC8282819; doi:10.1038/s41467-021-24379-z)
Supplement: Supplementary file 4 — Reporting Summary [file 41467_2021_24379_MOESM4_ESM.pdf]

nature research

Corresponding author(s): Zhenbiao Yang, Xiaohua Huang

Last updated by author(s): Jun 5, 2021

Reporting Summary

Nature Research wishes to improve the reproducibility of the work that we publish. This form provides structure for consistency and transparency in reporting. For further information on Nature Research policies, see our Editorial Policies and the Editorial Policy Checklist.

Statistics

For all statistical analyses, confirm that the following items are present in the figure legend, table legend, main text, or Methods section.

n/a	Confirmed	
☐	☒	The exact sample size (n) for each experimental group/condition, given as a discrete number and unit of measurement
☐	☒	A statement on whether measurements were taken from distinct samples or whether the same sample was measured repeatedly
☐	☒	The statistical test(s) used AND whether they are one- or two-sided<br>*Only common tests should be described solely by name; describe more complex techniques in the Methods section.*
☒	☐	A description of all covariates tested
☒	☐	A description of any assumptions or corrections, such as tests of normality and adjustment for multiple comparisons
☐	☒	A full description of the statistical parameters including central tendency (e.g. means) or other basic estimates (e.g. regression coefficient) AND variation (e.g. standard deviation) or associated estimates of uncertainty (e.g. confidence intervals)
☐	☒	For null hypothesis testing, the test statistic (e.g. F, t, r) with confidence intervals, effect sizes, degrees of freedom and P value noted<br>*Give P values as exact values whenever suitable.*
☒	☐	For Bayesian analysis, information on the choice of priors and Markov chain Monte Carlo settings
☒	☐	For hierarchical and complex designs, identification of the appropriate level for tests and full reporting of outcomes
☒	☐	Estimates of effect sizes (e.g. Cohen's d, Pearson's r), indicating how they were calculated

Our web collection on statistics for biologists contains articles on many of the points above.

Software and code

Policy information about availability of computer code

Data collection	MassHunter, Qtegra™ Intelligent Scientific Data Solution™ and Roche lightcycler 96 software was used to collect the LC-MS, ICP-MS and qPCR data, respectively.
Data analysis	ImageJ, MassHunter, Qtegra™ Intelligent Scientific Data Solution™ and Roche lightcycler 96 software was used to collect/analyze the CLSM, LC-MS, ICP-MS and qPCR data, respectively.

For manuscripts utilizing custom algorithms or software that are central to the research but not yet described in published literature, software must be made available to editors and reviewers. We strongly encourage code deposition in a community repository (e.g. GitHub). See the Nature Research guidelines for submitting code & software for further information.

Data

Policy information about availability of data

All manuscripts must include a data availability statement. This statement should provide the following information, where applicable:

- Accession codes, unique identifiers, or web links for publicly available datasets
- A list of figures that have associated raw data
- A description of any restrictions on data availability

The source data underlying Figs. 1b, e and g, 2b and d, 3b-d, 4e, Supplementary Figs 1b and d, 2b, 3c, 4-6, 7b and d, 8b and d, 9, 10b, and Supplementary Tables 1-4 are provided as a Source Data file. All other data included in the manuscript and/or supplemental materials are available from the corresponding authors upon reasonable request. Source data are provided with this paper.

Field-specific reporting

Please select the one below that is the best fit for your research. If you are not sure, read the appropriate sections before making your selection.

- [x] Life sciences
- [ ] Behavioural & social sciences
- [ ] Ecological, evolutionary & environmental sciences

For a reference copy of the document with all sections, see nature.com/documents/nr-reporting-summary-flat.pdf

Life sciences study design

All studies must disclose on these points even when the disclosure is negative.

Sample size	Sample size was determined based on past experience and typical sample sizes reported in related scientific literature. No statistical tests were performed to determine sample size before experiments. The exact sample size (n) and p-value are reported for each experiment.
Data exclusions	No data was excluded from the anaysis.
Replication	All experiments, unless specified, were conducted for six independent measurements (six times) and each treatment group in each measurement contained three replicates.
Randomization	The allocation of samples into experimental groups was random.
Blinding	The investigators were blinded to group allocation during data analysis.

Reporting for specific materials, systems and methods

We require information from authors about some types of materials, experimental systems and methods used in many studies. Here, indicate whether each material, system or method listed is relevant to your study. If you are not sure if a list item applies to your research, read the appropriate section before selecting a response.

Materials & experimental systems

n/a	Involved in the study	
[ ]	[x]	Antibodies
[x]	[ ]	Eukaryotic cell lines
[x]	[ ]	Palaeontology and archaeology
[x]	[ ]	Animals and other organisms
[x]	[ ]	Human research participants
[x]	[ ]	Clinical data
[x]	[ ]	Dual use research of concern

Methods

n/a	Involved in the study	
[x]	[ ]	ChIP-seq
[x]	[ ]	Flow cytometry
[x]	[ ]	MRI-based neuroimaging

Antibodies

Antibodies used	Anti-plant actin mouse monoclonal antibody (A01050, http://www.abbkine.cn/product/a01050) was purchased from Abbkine Scientific Co. Ltd. (Wuhan, China). Goat anti-mouse IgG H&L (HRP) (ab205719, https://www.abcam.cn/goat-mouse-igg-hl-hrp-ab205719.html) and HRP anti-GFP antibody (ab6663, https://www.abcam.cn/hrp-gfp-antibody-ab6663.html) were purchased from Abcam Co. Ltd. (Shanghai, China).
Validation	The validation statements of each primary antibody are available on the manufacturer's website (http://www.abbkine.cn/product/a01050, https://www.abcam.cn/goat-mouse-igg-hl-hrp-ab205719.html and https://www.abcam.cn/hrp-gfp-antibody-ab6663.html)
